# Analysis of serum microRNA expression in male workers with occupational noise-induced hearing loss

**DOI:** 10.1590/1414-431X20176426

**Published:** 2018-01-11

**Authors:** Y.H. Li, Y. Yang, Y.T. Yan, L.W. Xu, H.Y. Ma, Y.X. Shao, C.J. Cao, X. Wu, M.J. Qi, Y.Y. Wu, R. Chen, Y. Hong, X.H. Tan, L. Yang

**Affiliations:** 1 School of Medicine, Hangzhou Normal University, Hangzhou, Zhejiang, China; 2 College of Life Sciences, Shihezi University, Shihezi, Xinjiang, China; 3 Hangzhou Hospital for the Prevention and Treatment of Occupational Diseases, Hangzhou, Zhejiang, China

**Keywords:** microRNA, Noise-induced hearing loss, Serum, miR-1229-5p, MAPK1

## Abstract

Occupational noise-induced hearing loss (ONIHL) is a prevalent occupational disorder that impairs auditory function in workers exposed to prolonged noise. However, serum microRNA expression in ONIHL subjects has not yet been studied. We aimed to compare the serum microRNA expression profiles in male workers of ONIHL subjects and controls. MicroRNA microarray analysis revealed that four serum microRNAs were differentially expressed between controls (n=3) and ONIHL subjects (n=3). Among these microRNAs, three were upregulated (hsa-miR-3162-5p, hsa-miR-4484, hsa-miR-1229-5p) and one was downregulated (hsa-miR-4652-3p) in the ONIHL group (fold change >1.5 and P_bon_ value <0.05). Real time quantitative PCR was conducted for validation of the microRNA expression. Significantly increased serum levels of miR-1229-5p were found in ONIHL subjects compared to controls (n=10 for each group; P*<*0.05). A total of 659 (27.0%) genes were predicted as the target genes of miR-1229-5p. These genes were involved in various pathways, such as mitogen-activated protein kinase (MAPK) signaling pathway. Overexpression of miR-1229-5p dramatically inhibited the luciferase activity of 3′ UTR segment of MAPK1 (P<0.01). Compared to the negative control, HEK293T cells expressing miR-1229-5p mimics showed a significant decline in mRNA levels of MAPK1 (P<0.05). This preliminary study indicated that serum miR-1229-5p was significantly elevated in ONIHL subjects. Increased miR-1229-5p may participate in the pathogenesis of ONIHL through repressing MAPK1 signaling.

## Introduction

Occupational noise-induced hearing loss (ONIHL), characterized by auditory function impairments after long-term and persistent exposure to excessive noise, is a common occupational disease (1,2). According to a recent systematic review, the prevalence of ONIHL in noise-exposed workers ranges from 7 to 21% (3). Although the pathogenesis of ONIHL has not yet been fully described, it is suggested that this disorder results from both genetic and environmental factors (4,5). Individual variations in the susceptibility to noise exposure might be associated with genetic factors (6).

MicroRNAs are a family of short, single-stranded, non-coding RNAs that play pivotal roles in modulating gene expression by targeting mRNAs for cleavage or translational repression (7). The use of circulating microRNAs, from full blood, serum or plasma, as predictive biomarkers, have been implicated in several human disorders, including cancer (8), neurological disorders (9), cardiovascular diseases (10), and infectious diseases (11). In a previous study, Ding et al. (12) compared the plasma level of microRNAs between ONHIL subjects and controls, and identified several upregulated (miR-16-5p, miR-24-3p, miR-185-5p and miR-451a) and downregulated (miR-24, miR-185-5p and miR-451a) microRNAs in ONIHL subjects. Nevertheless, the expressions of differential microRNAs in the serum of ONIHL individuals have not yet been studied.

The aim of this preliminary study based on a small cohort of ONIHL was to compare the serum expression profiles of microRNAs between ONIHL subjects and controls using microRNA microarray assay.

## Material and Methods

### Ethics statement

Ethical approval for this study was granted by the Ethics Committee of Hangzhou Hospital for the Prevention and Treatment of Occupational Diseases, China. Subjects were recruited after giving written informed consent.

### Study design and subjects

Ten workers with ONIHL and 10 noise-exposed individuals with normal hearing (controls) who received their occupational health examination at Hangzhou Hospital for the Prevention and Treatment of Occupational Diseases were enrolled in this study. Participants who worked in different industries were randomly selected. Because most individuals working under the noise-exposed environment are male, we analyzed male subjects only. Self-reported work duration per day was recorded. Two groups of cohorts were matched in age, gender and work duration. All subjects received pure tone audiometry. Average level of binaural high-frequency hearing threshold (at 3, 4, 6 kHz), as well as the weighted value of monaural threshold at speech frequencies (0.5, 1, 2 kHz) and high frequency (4 kHz) were calculated. According to the national occupational health standards of the People’s Republic of China for the diagnosis of occupational noise-induced deafness in 2014, ONIHL was defined in those with average level of binaural high-frequency hearing threshold ≥40 dB, and the weighted value of monaural threshold of the better ear ≥26 dB. Individuals with normal hearing were defined as those with binaural hearing threshold at all frequencies <26 dB.

Inclusion criteria were as follows: 1) age ≥30 years and <45 years; 2) more than 1 year work experience in a noisy environment; 3) male. The industries of ONIHL workers included machinery (8 cases), food (1 case), and chemical industry (1 case). The industries of control subjects include machinery (4 cases), manufacturing (4 cases), energy (1 case), and printing (1 case). The demographic and clinical characteristics of subjects are listed in Table 1. Blood was collected and centrifuged at 1000 *g* for 10 min at 4°C. Blood serum was then collected and stored at -80°C until use. The blood samples of three ONIHLs and three controls were used for microarray analysis. The serum levels of candidate microRNAs in all blood samples were verified by real time quantitative PCR (qPCR).

**Table 1.** Demographic and clinical characteristics of subjects used for microarray analysis and PCR experiments.

	Microarray analysis			PCR analysis		
	Control	ONIHL	P value	Control	ONIHL	P value
Number of subjects	3	3		7	7	
Gender (male/female)	3/0	3/0		7/0	7/0	
Age (years)	36.7±6.4	43.3±0.6	0.081	41.6±1.1	42.4±1.3	0.208
Average level of binaural high-frequency hearing threshold (dB)	25.0±0.0	60.3±0.6	**<0.001**	25.0±0.0	63.3±2.4	**<0.001**
Weighted value of monaural threshold of the better ear (dB)	25.0±0.0	30.7±1.3	**0.013**	25.0±0.0	31.9±1.3	**<0.001**
Working duration (h/day)	8.5±0.9	8.3±0.6	0.795	9.4±1.7	9.0±1.5	0.607

PCR: polymerase chain reaction; ONIHL: occupational noise-induced hearing loss. Data are reported as number of cases or means±SD. P values compared to control are reported (*t*-test).

### Extraction and purification of microRNA

MicroRNA was extracted and purified from blood serum of subjects using mirVanaTM PARISTM (Cat #AM1556) kit according to the manufacturer’s instructions (Ambion, USA). The quality and quantity of the isolated microRNAs were measured using an ND-2000 NanoDrop spectrophotometer (ThermoScientific, USA) and Agilent Bioanalyzer 2100 (Agilent Technologies, USA).

### microRNA microarray analysis

Microarray hybridization experiment was conducted for profiling differentially expressed miRNAs between the two groups. Each slide was hybridized with 100 ng Cy3-labeled RNA using miRNA Complete Labeling and Hyb Kit (Cat #5190–0456, Agilent Technologies) in hybridization oven (Cat #G2545A, Agilent Technologies) at 55°C at a rotation speed of 20 rpm for 20 h. After hybridization, slides were washed in staining dishes (Cat #121, Thermo Shandon, USA) with Gene Expression Wash Buffer Kit (Cat #5188–5327, Agilent Technologies). Slides were scanned by Agilent Microarray Scanner (Cat #G2565CA, Agilent Technologies) and analyzed by Feature Extraction software 10.7 (Agilent Technologies) with default settings. Raw data were normalized by Quantile algorithm, Gene Spring Software 12.6 (Agilent Technologies).

### Real time qPCR for evaluating serum microRNA level

Real time qPCR was performed to validate the expression levels of the candidate microRNAs screened from the microarray assay. PCR primers were synthesized by Sangon Biotech., China. microRNA was subjected to a poly (A)-tailed reverse transcription, followed by PCR amplification in a reaction mixture containing 2.8 µL cDNA template, 0.2 µL forward primer, 0.2 µL reverse primer, 5 µL 2× miRcute miRNA Premix and 1.8 µL RNase-free ddH_2_O to a final volume of 10 µL. PCR reaction was performed on PikoReal Thermal Cycler 96-well system (TCP0096) with the following procedures: a 2-min denaturation at 94°C, 45 cycles of PCR amplification at 94°C for 20 s, and 60°C for 34 s. Each sample was tested in triplicate. The expression level of each microRNA in ONIHL group was individually normalized to control. Data were calculated by 2-ΔΔCT method (13).

### Cell culture

Human embryonic kidney (HEK293T) cells were cultured in Dulbecco’s Modified Eagle Medium (DMEM) containing 10% fetal bovine serum (FBS), 100 U/mL penicillin and 100 µg/mL streptomycin at 37°C in a 5% CO_2_ incubator.

### Luciferase dual reporter assay

The multiple cloning sites of GV272 vector (Shanghai Genechem Co., Ltd., China) were digested by XbaI/XbaI restriction enzymes, and the 3′ untranslated regions (UTR) segment of MAPK1 (NM_002745) or MAPK1-mutant (position 3047–3053, CUACCCA→AGCAAAC) was inserted into the vector to yield 3′ UTR MAPK1 or 3′ UTR-MU MAPK1. The recombinant products were verified by DNA sequencing. GV272 empty vector was utilized as negative control (3′ UTR-NC MAPK1). hsa-miR-146b and its target gene TRAF6 were recruited as positive control (14). HEK293T cells were seeded onto a 24-well plate. On the next day, cells were co-transfected with 3′ UTR luciferase plasmid (0.1 μg), microRNA plasmid (0.4 μg) and Renilla plasmid (0.02 μg) using X-tremeGENE HP DNA Transfection Reagent (Roche). Six experimental groups were set as follows: 3′ UTR-NC MAPK1+miR-1229-5p-NC; 3′ UTR-NC MAPK1+miR-1229-5p; 3′ UTR MAPK1+miR-1229-5p-NC; 3′ UTR MAPK1+miR-1229-5p; 3′ UTR-MU MAPK1+miR-1229-5p-NC; 3′ UTR-MU MAPK1+miR-1229-5p. Cells transfected with 3′ UTR TRAF6+miR-146b-NC or 3′ UTR TRAF6+miR-146b were used as positive control. Forty-eight hours after transfection, the luciferase activity was measured using Dual-Luciferase® Reporter Assay System according to manufacturer’s instructions (Promega, USA) on a microplate reader (Tecan infinite, Switzerland). The relative luciferase activity was calculated by normalizing the ratio of Firefly/Renilla luciferase to the negative control. In brief, the relative luciferase activity of 3′ UTR-NC MAPK1+miR-1229-5p, 3′ UTR MAPK1+miR-1229-5p, 3′ UTR-MU MAPK1+miR-1229-5p was normalized to 3′ UTR-NC MAPK1+miR-1229-5p-NC, 3′ UTR MAPK1+miR-1229-5p-NC, and 3′ UTR-MU MAPK1+miR-1229-5p-NC, respectively.

### microRNA mimics transfection

One day prior to transfection, cells were seeded onto a 6-well plate at a density of 4×105 cells/well. Before transfection, culture medium was replaced with 2 mL of serum- and antibiotic-free DMEM medium. Lipofectamine™ 2000 was used for transfection of hsa-miR-1229-5p mimics. Briefly, 7.5 µL Lipofectamine™ 2000 was gently mixed with 100 µL Opti-MEM culture medium and incubated for 5 min at room temperature. Then, 5.5 µL hsa-miR-1229-5p mimics were added into 100 µL Opti-MEM culture medium and incubated for 5 min. After that, the solution containing microRNA mimics was mixed with transfection reagent and incubated for another 20 min, followed by adding onto cell culture medium. Negative control cells were treated with transfection reagent in the absence of microRNA mimics. After 4–6 h of transfection, the medium was replaced with fresh culture medium. Cells were incubated at 37°C for another 48 h until RNA extraction.

### Real time qPCR for determining the mRNA level of target gene

Total RNA was extracted from HEK293T cells and reversely transcribed into cDNA using FastQuant RT Kit (China). Real time PCR reaction solution contains cDNA template, 0.3 μL forward primer (0.3 μM), 0.3 μL reverse primer (0.3 μM), 5 μL 2×SuperReal PreMix Plus and RNase-Free ddH_2_O to a final volume of 10 μL. PCR reaction was carried out using the following procedures: a 15-min denaturation at 95°C, 40 cycles of PCR amplification at 95°C for 10 s and 60°C for 31 s. Each sample was tested in triplicate. Data were calculated by 2-ΔΔCT method (13).

### Statistical analysis

Statistical analysis was performed using the IBM SPSS Statistics 20 for Windows (USA). Data are reported as the number (percentage) of cases or means±SD. Differences between two experimental groups were compared using Student’s *t*-test. Bonferroni correction was utilized for multiple hypothesis testing. A P value <0.05 was considered to be statistically significant.

## Results

### Demographic and clinical characteristics of subjects

To identify the differentially expressed microRNAs in the blood serum between controls and ONIHL subjects, microarray analysis was utilized. Three male individuals from each group were recruited in this experiment. There was no significant difference in the gender, age, or daily work duration between the two groups (P>0.05, Table 1). Compared with controls, ONIHL subjects had an increased average level of binaural high-frequency hearing threshold (60.3±0.6 dB *vs.* 25.0±0.0 dB; P<0.001) and an elevated weighted value of monaural threshold of the better ear (30.7±1.3 dB *vs* 25.0±0.0 dB; P=0.013).

### microRNA profiling

**Figure 1.**
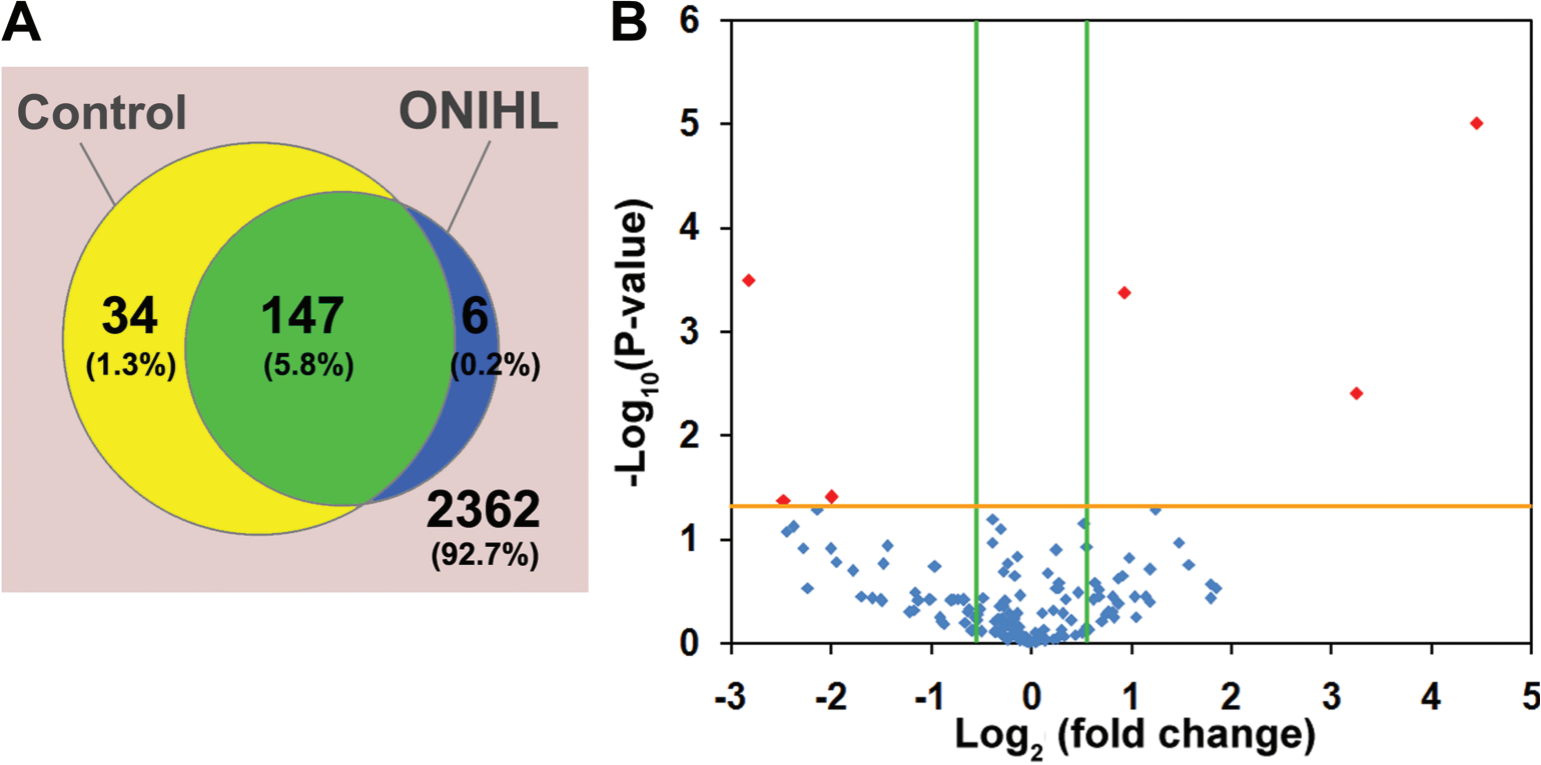
MicroRNA profile analyzed by microarray. *A*, microRNA profile. Among 2549 tested microRNAs, 2362 (92.7%) were not expressed in either group; 147 (5.8%) microRNAs were commonly expressed in both groups; while 34 (1.3%) and 6 (0.2%) microRNAs were found to be exclusively expressed in controls and ONIHL subjects, respectively. *B*, Volcano plots representing the differentially expressed microRNAs between occupational noise-induced hearing loss (ONIHL) and control subjects. The X-axis represents the log2-transformed fold change compared with controls; the Y-axis represents –log10 (P values) obtained from the Student's *t*-test. Dots on the right side of the green line represent the upregulated microRNAs with a fold change >1.5, and those located on the left side of the green line represent downregulated microRNAs with fold change >1.5 in ONIHL subjects when compared to those of controls. P value threshold was set at 0.05 (orange line). Those differentially expressed microRNAs, with a fold change >1.5 and P<0.05, are indicated in red.

MicroRNA microarray assay showed that among 2549 tested microRNAs, 2362 (92.7%) were not expressed in either groups, while 34 (1.3%) and 6 (0.2%) microRNAs were found to be exclusively expressed in controls and ONIHL subjects, respectively (Figure 1A). Besides, 147 (5.8%) microRNAs were commonly expressed in both groups.

### Identification of differentially expressed serum microRNAs

There were seven differentially expressed microRNAs between control and ONIHL subjects, with a fold change >1.5 and P<0.05. Among these microRNAs, three were upregulated (hsa-miR-3162-5p, hsa-miR-4484, hsa-miR-1229-5p) and four were downregulated (hsa-miR-6752-3p, hsa-miR-6824-3p, hsa-miR-4769-3p, hsa-miR-4652-3p) in ONIHL subjects (Figure 1B). After Bonferroni correction, we identified four differentially expressed microRNAs between the two groups, namely hsa-miR-3162-5p, hsa-miR-4484, hsa-miR-1229-5p, and hsa-miR-4652-3p (Table 2).

**Table 2.** Differentially expressed serum microRNAs between ONIHL patients and controls.

	Fold change	P/P_bon_
Upregulated microRNAs		
hsa-miR-3162-5p	21.89	**9.7E-6/6.8E-5**
hsa-miR-4484	9.51	**0.004/0.027**
hsa-miR-1229-5p	1.91	**4.2E-4/0.003**
Downregulated microRNAs		
hsa-miR-6752-3p	0.25	0.039/0.276
hsa-miR-6824-3p	0.25	0.039/0.276
hsa-miR-4769-3p	0.18	0.042/0.296
hsa-miR-4652-3p	0.14	**3.2E-4/0.002**

ONIHL: occupational noise-induced hearing loss. P_bon_ value was calculated by the Bonferroni correction using the following equation: P_bon_ value = P value × 7 (7 microRNAs).

**Figure 2.**
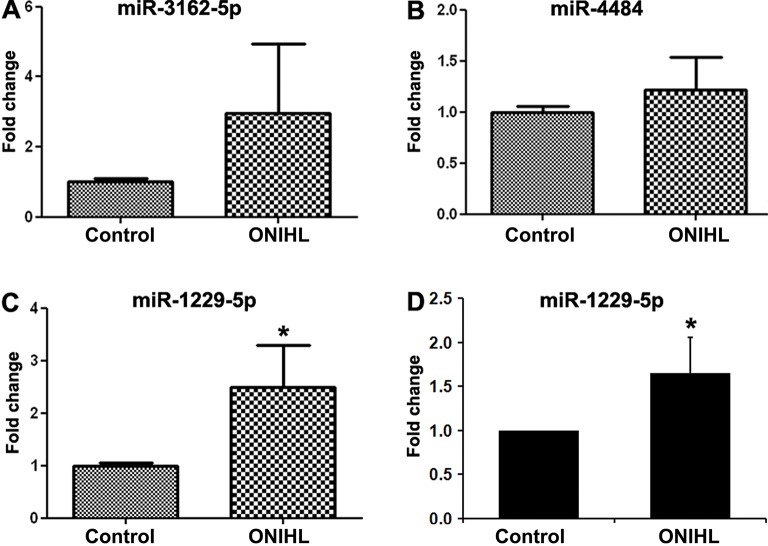
The differentially expressed miR-3162-5p (*A*), miR-4484 (*B*), and miR-1229-5p (*C*, *D*) in the blood serum of occupational noise-induced hearing loss (ONIHL) patients and controls were verified using real time qPCR. *A*–*C*, Blood serum samples were derived from those used for microarray analysis (n=3 for each group). *D*, Blood serum samples were derived from subjects that did not receive microarray analysis (n=7 for each group). Data are reported as means±SD. *P*<*0.05 compared with control (*t*-test).

As fold changes of upregulated microRNAs were greatly higher than that of the downregulated ones, the levels of the differentially expressed miR-3162-5p, miR-4484, and miR-1229-5p in the blood serum of ONIHL subjects and controls were verified using real time qPCR. Consistent with the microarray results, the serum levels of miR-3162-5p, miR-4484, and miR-1229-5p were elevated in ONIHL subjects when compared to that in controls (Figure 2A-C). However, it should be noted that a significant increase of serum expression was only detected for miR-1229-5p (P*<*0.05). Seven ONIHL subjects and seven controls, the samples of whom were not tested in microarray assay, were recruited for further analysis. Likewise, an increased serum level of miR-1229-5p was found in ONIHL group as compared with controls (P*<*0.05, Figure 2D).

### Functional annotation of putative target genes of hsa-miR-1229-5p

**Figure 3.**
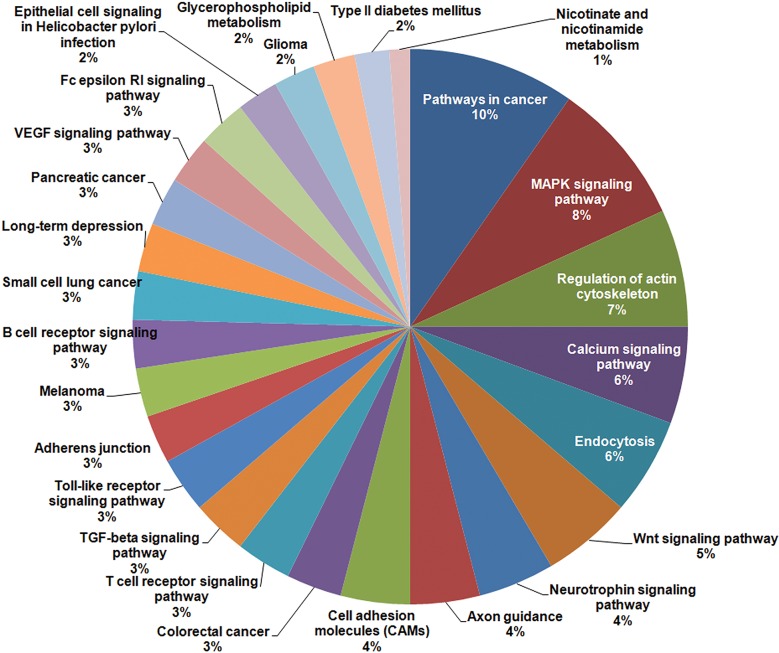
Pie chart of the predicted target genes of miR-1229-5p involved in various pathways. Data were analyzed by KEGG pathways using DAVID tool.

We next searched the predicted targets of miR-1229-5p on the website of TargetScanHuman (http://www.targetscan.org/vert_71/). A total of 2660 transcripts, containing 3157 sites, were detected. Functional annotation of the predicted genes was analyzed by gene ontology (GO) and Kyoto Encyclopedia of Genes and Genomes (KEGG) pathways using the Database for Annotation, Visualization and Integrated Discovery (DAVID 6.7; http://david.abcc.ncifcrf.gov) tools (15,16). GO analysis revealed that the predicted target genes of hsa-miR-1229-5p were involved in a great number of biological processes (e.g., cellular, metabolic, biosynthetic, localization, developmental, etc.), cellular components (plasma membrane, non-membrane-bounded organelle, nuclear lumen, cell fraction, endoplasmic reticulum, etc.) and molecular functions (ion binding, nucleotide binding, ATP binding, transcription regulator activity, protein kinase activity, etc.) (Table 3). In addition, 659 (27.0%) of the predicted target genes of miR-1229-5p were involved in various pathways, such as pathways in cancer, mitogen-activated protein kinase (MAPK) signaling pathway, regulation of actin cytoskeleton, calcium signaling pathway, endocytosis, etc. (Figure 3).

**Table 3.** Gene ontology analysis of the predicted target genes of hsa-miR-1229-5p.

Term	Count	Percentage (%)	P value	Benjamini
Biological processes	1795	73.7		
Cellular process	1505	61.8	P<0.05	P<0.05
Metabolic process	1058	43.4	P<0.05	P=0.56
Biosynthetic process	504	20.7	P<0.05	P=0.66
Localization	456	18.7	P<0.05	P=0.30
Developmental process	450	18.5	P<0.05	P=0.67
Cellular components	1632	67.0		
Plasma membrane	530	21.7	P<0.05	P=0.29
Non-membrane-bounded organelle	353	14.5	P=0.093	P=0.73
Nuclear lumen	203	8.3	P=0.088	P=0.73
Cell fraction	162	6.6	P<0.05	P=0.48
Endoplasmic reticulum	155	6.4	P<0.05	P=0.18
Molecular functions	1732	71.1		
Ion binding	631	25.9	P<0.05	P=0.27
Nucleotide binding	351	14.4	P<0.05	P=0.10
ATP binding	223	9.2	P<0.05	P=0.73
Transcription regulator activity	221	9.1	P=0.075	P=0.88
Protein kinase activity	95	3.9	P=0.06	P=0.86

Gene Ontology analysis contains three functional classes, namely biological processes, cellular components and molecular functions. The number or percentage of genes within categories ranked in the top five of each class is listed. Benjamini: Benjamini-Hochberg corrected P value.

### Identification of a new target of miR-1229-5p, MAPK1

**Figure 4.**
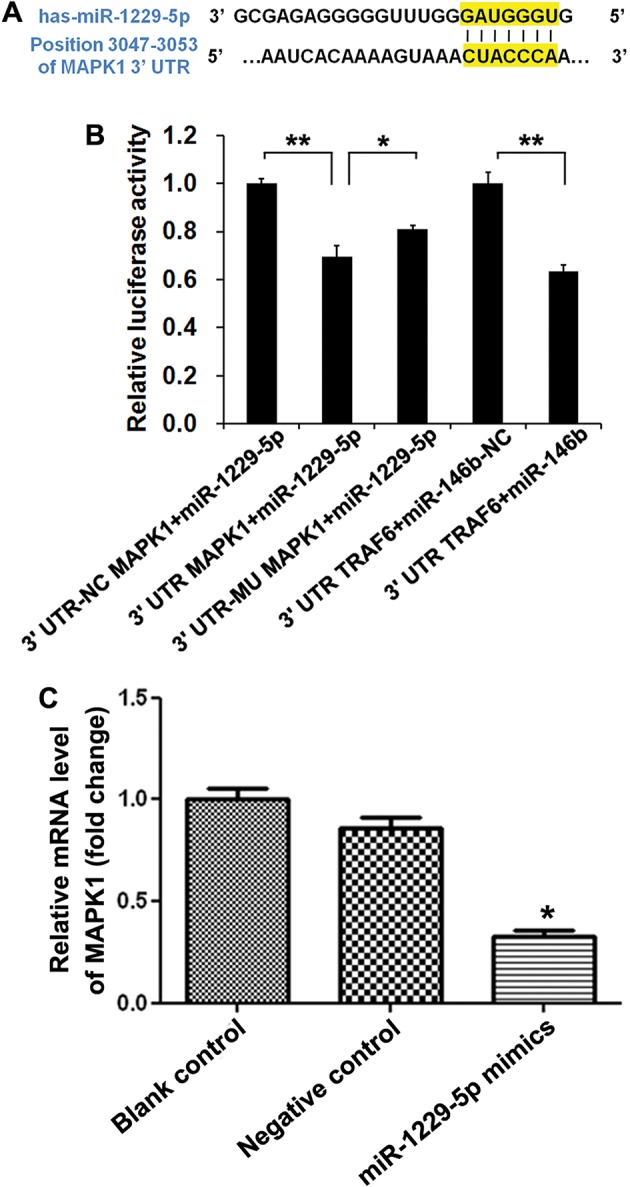
*A*, Sequence alignment of miR-1229-5p and its target sites in 3′ UTR segments of human MAPK1 gene. *B*, Relative luciferase activity. Cells were co-transfected with 3′ UTR luciferase plasmid, microRNA plasmid and Renilla plasmid. Luciferase activity was measured using Dual-Luciferase® Reporter Assay System 48 h after transfection. The relative luciferase activity was calculated against the negative control. *P*<*0.05, **P*<*0.01 (*t*-test). *C*, Cells were transfected with miR-1229-5p mimics using Lipofectamine™ 2000 transfection reagent. Cells exposed to transfection reagent or not transfected were used as negative control and blank control, respectively. Forty-eight hours after transfection, the mRNA level of MAPK1 was examined using real time qPCR. The mRNA expression of blank control was set as 1 and the relative fold changes of negative control and miR-1229-5p mimics are reported. Data are reported as means±SD. *P*<*0.05 compared with negative control (*t*-test).

We found that the 3′ UTR segment of human MAPK1 gene contains a sequence that is complementary to nucleotides of hsa-miR-1229-5p (Figure 4A). To understand the regulatory effect of hsa-miR-1229-5p on MAPK1, luciferase dual reporter assay was applied. The has-miR146b has been reported to target TRAF6 (14). As shown by Figure 4B, co-transfecting miR-146b and TRAF6 into HEK293T cells significantly reduced the luciferase activity when compared to the negative control (P*<*0.01), indicating that the luciferase dual reporter assay works. In addition, overexpression of miR-1229-5p dramatically inhibited the activity of 3′ UTR segment of MAPK1 as compared with the negative control (P*<*0.01). In contrast, mutation in 3′ UTR segment of MAPK1 reversed the downregulated luciferase activity induced by miR-1229-5p (P*<*0.05). Finally, miR-1229-5p mimics were synthesized and transfected into HEK293T cells. Real time qPCR analysis demonstrated that, compared to the negative control, cells expressing miR-1229-5p mimics showed a significant decline in the mRNA level of MAPK1 (P*<*0.05) (Figure 4C).

## Discussion

Hearing loss is associated with a variety of genetic and environmental factors, including male gender, age, and noise exposure (3). In this study, 10 controls and 10 ONIHL subjects were enrolled. Note that only male participants were recruited for analysis, because most of the noise-exposed industrial workers are male.

MicroRNA analysis identified four differentially expressed microRNAs in serum between controls and ONIHL subjects. These differentially expressed microRNAs in blood circulation were further verified by real time qPCR. Among these differentially expressed microRNAs, increased serum levels of miR-3162-5p and miR-4484 have been reported to be associated with the enhanced lymph node metastasis of cervical squamous cell carcinoma (17). However, the serum expressions of other microRNAs and their relevant functions have not yet been studied. Ding et al. (12) compared the plasma level of microRNAs between ONIHL subjects and controls and identified 73 differentially expressed (39 upregulated and 34 downregulated) microRNAs, among which six microRNAs, different from those identified in our study, were selected for validation analysis. Such discrepancy might be due to the different profiles of serum or plasma as the source of microRNA.

A significant elevation of serum miR-1229-5p was detected in ONIHL subjects when compared to that in controls, indicating miR-1229-5p may be involved in the pathogenesis of ONIHL. We also analyzed the predicted targets of miR-1229-5p. By using luciferase dual reporter assay, MAPK1 was identified as a new target of miR-1229-5p. A comprehensive network and pathway analysis indicated that the MAPK3/MAPK1 MAP kinase serves as one of the crucial nodal molecules in regulating human genetic deafness (18), highlighting the close association between MAPK signaling pathway and hearing loss. Using a rodent model of noise-induced hearing loss, Alagramam et al. (19) demonstrated that MAPK signaling pathway was activated after noise exposure. *MAPK1*, known as ERK2, plays multifactorial roles in mediating cell survival, proliferation, differentiation, and death (20). The ERK1/2 signaling kinases are potential modulators for the genetic hearing loss (18). In cultured cochlear explants, aminoglycoside incubation results in ERK1/2 activation in supporting cells, which promotes the death of surrounding inner hair cell (21). Moreover, it has been reported that MAPK1 contributes to the modulation of inner hair cell survival and decreases the susceptibility to noise-induced hearing loss in mice (22). Therefore, it is possible that MAPK1 signaling in inner hair cells acts as a self-defensive mechanism against noise exposure. miR-1229-5p may contribute to the pathogenesis of ONIHL through producing a post-transcriptional repression of MAPK1. Among cells in the organ of Corti, the outer hair cells are known to be most vulnerable to over-loaded sound (23). However, little is known about the pro-survival or pro-death role of MAPK1 in regulating the outer hair cells. Besides, the mutation of MAPK1 has been implicated in the tumorigenesis of cervical cancer and ovarian cancer (24,25), although the involvement of its mutation in hearing loss remains unknown. In our future study, the incidence of MAPK1 mutation and its influences on the miR-1229-5p expression will be explored.

Collectively, our current findings indicated that serum miR-1229-5p level may be a novel biomarker for predicting ONIHL. Evaluation of the circulating miR-1229-5p concentration may facilitate disease prevention and early interference of ONIHL. Moreover, miR-1229-5p may contribute to the pathogenesis of hearing loss possibly via its negative regulation of MAPK1. Nevertheless, our current study had several limitations. First, in this preliminary study, we aimed to understand the differences in the serum microRNA expression profiles between ONIHL and control subjects, so the sample size was small. Future studies will be carried out by enrolling more individuals with ONIHL and controls to confirm the clinical significance and diagnostic value of serum miR-1229-5p level. Second, the subjects in this study were all male. In future studies, female workers with ONIHL will also be included for understanding the correlation between miR-1229-5p expression and gender. Third, the crucial regulatory role of MAPK1 for ONIHL has not yet been fully elucidated. DNA methylation, mutation, as well as the downstream gene expression of MAPK1 will be further examined. We could also not rule out the possibility that miR-1229-5p may participate in ONIHL pathogenesis through mediating other signaling pathways.
